# Comparison of various texture classification methods using multiresolution analysis and linear regression modelling

**DOI:** 10.1186/s40064-015-1631-1

**Published:** 2016-01-20

**Authors:** S. Dhanya, V. S. Kumari Roshni

**Affiliations:** Federal Institute of Science And Technology, Kerala, India; Centre for Development of Advanced Computing (CDAC), Bangalore, India

**Keywords:** Dual tree complex wavelet packet transform, Discrete wavelet packet transform, Discrete wavelet transform, Linear regression, Texture classification

## Abstract

Textures play an important role in image classification. This paper proposes a high performance texture classification method using a combination of multiresolution analysis tool and linear regression modelling by channel elimination. The correlation between different frequency regions has been validated as a sort of effective texture characteristic. This method is motivated by the observation that there exists a distinctive correlation between the image samples belonging to the same kind of texture, at different frequency regions obtained by a wavelet transform. Experimentally, it is observed that this correlation differs across textures. The linear regression modelling is employed to analyze this correlation and extract texture features that characterize the samples. Our method considers not only the frequency regions but also the correlation between these regions. This paper primarily focuses on applying the Dual Tree Complex Wavelet Packet Transform and the Linear Regression model for classification of the obtained texture features. Additionally the paper also presents a comparative assessment of the classification results obtained from the above method with two more types of wavelet transform methods namely the Discrete Wavelet Transform and the Discrete Wavelet Packet Transform.

## Background

Image classification refers to the classification of images based on the visual content. It includes object recognition and scene classification. Important applications include industrial and biomedical surface inspection, for example for defects and disease, ground classification and segmentation of satellite or aerial imagery, segmentation of textured regions in document analysis, and content-based access to image databases. Textures in images provide information about spatial arrangement of intensities or colour in an image. They play an important role in many image classification tasks. A fundamental issue in texture based classification tasks is how to effectively characterize the texture from the derived features. Much research has occurred in this area during the last decade (Rui et al. 1999; Randen and Husøy 1999; Zhang and Tan 2002; Chen and Chen 1999; Haralick et al. 1973; Chen and Pavlidis 1983). The traditional approaches, focusing on the analysis of spatial relations between neighborhood pixels in a small region, included Gray Level Co-occurrence Matrix (GLCM) (Kashyap and Chellappa 1983; Unser 1986), second order gray level statistics (Unser 1995),and Gauss- Markov random field (Fernandez 2008). These methods perform best on micro-textures. Spectral histogram is yet another commonly used texture classification method (Liu and Wang 2003). It is based on local spatial/frequency information, which provides a unified texture feature.

Extensive research has demonstrated that classification based on multiresolution analysis methods resembling the human vision system provides better performance. Hence these methods are widely used for the classification of textures. Most commonly employed multiresolution analysis techniques include the Gabor transform (Grigorescu et al. 2002) and the wavelet transform (Van de Wouwer et al. 1999; Huang and Aviyente 2008).

In these methods the texture image is transformed by the use of the respective transform to local spatial/frequency representation by wrapping the image with appropriate band-pass filters tuned to specific parameters. In the case of Gabor transform, features such as Gabor energy, complex moments, and grating cell operator are considered to characterize the texture feature while in wavelet transform analysis (Van de Wouwer et al. 1999; Huang and Aviyente 2008; Ma and Manjunath 1995; Hackmack et al. 2012; Wang and Yong 2008) the wavelet coefficients itself serve the purpose of characterizing the texture features. In (Selesnick et al. 2005) the dual-tree complex wavelet transform is used to extract information on different spatial scales from structural MRI data and show its relevance for disease classification.

In this paper we apply linear regression modelling to analyse the correlation between texture samples at various frequencies, facilitated through multiresolution analysis, for the efficient classification of textures. The basic algorithm is adopted from (Wang and Yong 2008). In this work we have experimented with different multiresolution analysis tools that are used for texture classification. In addition to the miltiresolution analysis tools such as the discrete wavelet transform and the discrete wavelet packet transform that are suggested in (Wang and Yong 2008), we have employed the dual tree complex wavelet packet transform, which is the novelty adopted in this work and the performance of the three methods is compared. Our experiments show that, in most of the cases, the discrete wavelet transform outperforms the discrete wavelet packet transform and dual tree complex wavelet packet transform in terms of classification rate.

This paper is organized as follows. “Texture classification using linear regression model” provides an overview of the application of multiresolution analysis tools (Rahman et al. 2011) and linear regression modelling (Kerns 2011) for analysing correlation between frequency channels for the classification of textures. “Texture classification algorithm” explains the methodology adopted for the classification phase. The experimental results and the performance comparison with the different multiresolution analysis tools are presented in “Experimental results”. Finally the conclusions are briefed in “Conclusion”.

## Texture classification using linear regression model

### Multiresolution analysis tools

The level of detail within an image varies from location to location. Finer resolution for analysis is required at regions where significant information is contained. Multiresolution representation of an image provides complete detail about the extent of information present at different locations. The main concept of multiresolution analysis is that for each vector space, there is another vector space of higher resolution until the final image is obtained. The basis of each of these vector spaces is the scale function. For textures, it provides scale invariant interpretation of a texture.

Different multiresolution analysis tools that are used for texture analysis in this work are the following:

1. Discrete wavelet transform (DWT)
2. discrete wavelet packet transform (DWPT)
3. Dual tree complex wavelet packet transform (DTCWPT).

Discrete wavelet transform (DWT) of an image provides both frequency and location information of the analyzed image. The 2-D wavelet transform is carried out by the tensor product of two 1-D wavelet base functions along the horizontal and vertical directions, and the corresponding filters can be expressed as h_LL_(k, l) = h(k)h(l), h_LH_(k, l) = h(k)g(l), h_HL_(k, l) = g(k)h(l) and h_HH_(k, l) = g(k)g(l). By convolving the given image with these four filters, we get four sub images and thus four channels. Further decomposition is performed only in the low—low frequency region.

For quasi-periodic signals such as speech signals and texture patterns, whose dominant frequency channels are located in the middle frequency region, DWT decomposition is proposed to be non-ideal (Chang and Kuo 1993). Discrete Wavelet Packet Transform (DWPT) which allows further decomposition in all frequency regions to obtain full decomposition, is ideally suited for mid frequency regions (Chang and Kuo 1993). Thus DWPT of an image can characterize the properties of an image in all frequency regions. Wavelet packets perform better in terms of fidelity of direction but not in terms of improved directionality. The high-pass coefficients will oscillate around singularities of the signal (Ana SOVIC–Damir SERSIC 2012).

**Fig. 1:**
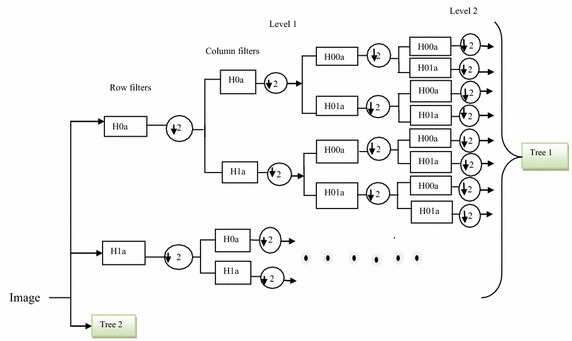
2-D dual tree complex wavelet packet decomposition

It is shown that the texture features which effectively define directional and spatial**/**frequency characteristics of the patterns lead to good texture analysis (Materka and Strzelecki 1998). In order to mitigate the limitations of DWT and DWPT, the complex wavelet transform can be used. To exploit the advantages of the complex transform of better shift invariance and directionality and that of the packet transform of better selectivity of decomposition, the combined transform or the dual tree complex wavelet packet transform (DTCWPT) is chosen in our work for further experiments. The dual-tree complex wavelet packet transform (DTCWPT) (Selesnick et al. 2005), involves two DWPT’s whose filters banks (FB) are designed so that the impulse responses of the first FB are approximately the discrete Hilbert transforms of those of the second FB as shown in Fig. 1. In this way it measures both the real (even) and the imaginary (odd) components of the input signal (hence the name complex wavelet transform).

For the three multiresolution analysis tools described above, the original image is first decomposed into four subimages by convolving the image with low pass and high pass filters both in the horizontal and vertical directions. The parent node and the four children nodes thus formed are named as O, A, B, C and D respectively. Each of the channels thus formed is given a channel number. This is to identify the frequency channels for further analysis of these channels in the experiments done. Table 1 shows the channel number assigned to the different channels formed as a result of decomposition using DWT and Table 2 shows the same for DWPT and DTCWPT.

**Table 1:** Channel number and naming used for DWT

Channel no	Name	Channel no	Name
1	OB	6	OAD
2	OC	7	OAAA
3	OD	8	OAAB
4	OAB	9	OAAC
5	OAC	10	OAAD

**Table 2:** Channel number and naming used for DWPT and DTCWPT

Channel no	Name	Channel no	Name
1	OAAA	33	OCAA
2	OAAB	34	OCAB
3	OAAC	35	OCAC
4	OAAD	36	OCAD
5	OABA	37	OCBA
6	OABB	38	OCBB
7	OABC	39	OCBC
8	OABD	40	OCBD
9	OACA	41	OCCA
10	OACB	42	OCCB
11	OACC	43	OCCC
12	OACD	44	OCCD
13	OADA	45	OCDA
14	OADB	46	OCDB
15	OADC	47	OCDC
16	OADD	48	OCDD
17	OBAA	49	ODAA
18	OBAB	50	ODAB
19	OBAC	51	ODAC
20	OBAD	52	ODAD
21	OBBA	53	ODBA
22	OBBB	54	ODBB
23	OBBC	55	ODBC
24	OBBD	56	ODBD
25	OBCA	57	ODCA
26	OBCB	58	ODCB
27	OBCC	59	ODCC
28	OBCD	60	ODCD
29	OBDA	61	ODDA
30	OBDB	62	ODDB
31	OBDC	63	ODDC
32	OBDD	64	ODDD

### Correlation between frequency channels

For explaining the correlation between frequency channels, decomposition using the Dual tree complex wavelet packet transform is considered here in lieu of the extensive directionality. In order to characterize the image texture, we can use the raw coefficients as such. But generally some measure derived from these values is taken as the texture feature, as handling the raw coefficients is difficult. Typical examples of these measures are mean, standard deviation, energy and so on. The energy distribution has important discriminatory properties for images as it reflects the distribution of energy along the frequency axis over scale and orientation and as such can be used as a feature for classification. In our experiments the energy values from the subimages are extracted using the mean of the magnitude of the subimage coefficients (Unser 1995). That is if (M, N) represents the size of the subimage I, and I(i, j) represents the subimage coefficient corresponding to (i, j), then its energy is given by the equation

1 $$ {\text{energy}}_{{{\text{I}}\left( {\text{M*N}} \right)}} = \frac{1}{\text{MN}}\sum_{\text{i = 1}}^{\text{M}} \left. {\sum_{\text{j = 1}}^{\text{N}} } \right|\left. {{\text{I}}\left( {{\text{i,}}\;{\text{j}}} \right)} \right| $$

Different samples of the same texture are taken to derive the inherent texture properties and DTCWPT decomposition is applied to each sample. Considering a three level decomposition, we get 64 real(even) and 64 imaginary(odd) components. If *ah* represents the real part and *ag* represents the imaginary part of the DTCWPT, the complex coefficients are given by *ah* + *j ag*. The magnitude of the complex coefficients is considered for further processing. Since the DTCWPT measures both the real and imaginary part of the input signal, it offers both magnitude and phase information. Hence it also characterizes the information in all frequency regions.

To arrive at the correlation between the derived channel pairs and thereby identify the significant channel pairs, we make use of a preprocessing algorithm which is detailed below.

The steps involved in the preprocessing algorithm is summarized as follows

[Input]: m samples of each texture
[Output]: channel pair list and energy matrix E for each texture

1. For each texture, take *m* samples and decompose each of the samples using three level DTCWPT.
2. For each sample of a given texture, we get n wavelet coefficients each for real and imaginary parts. From this we find the magnitude of the complex coefficients.
3. From the energy of each channel [to be found out using (1)], form the energy vector of size *n* for each of the texture samples.
4. Arrange the vectors to form energy matrix *E* of size *m x n*.
5. Find the correlation coefficient matrix *V* from *E* and arrange the channel pairs in the descending order of correlation coefficients.
6. Eliminate those channel pairs whose correlation coefficient is less than a predefined threshold value *T*.

Applying the DTCWPT 3-level decomposition, we get 64 frequency regions. Thus the energy matrix consists of energy vectors corresponding to 64 frequency channels of m samples. This energy matrix can be viewed in statistical perspective, as each frequency channel can be considered as a random variable and the energy values corresponding to each frequency channel can be considered as the values assigned to these random variables. From the energy matrix E_(m, 64)_, a correlation coefficient matrix V of size 64 × 64 is generated, where (i, j)th element in the matrix corresponds to the correlation coefficient between the i and jth channel. Here it is to be noted that the correlation between the same channel pairs will generate the value 1. So all the diagonal elements which have value 1 can be neglected. Due to the symmetric property of correlation matrix, the values below and to the left of the diagonal (lower triangle) will be same as the values above and to the right of the diagonal (upper triangle). Therefore the channel pairs corresponding to the upper or lower triangle need to be considered for further analysis.

For channel elimination and identification of the significant channel pairs, a threshold value T is to be selected. To establish a suitable value for T, we have experimented with different values of thresholds and sample numbers to find an optimum value. We have used 20 brodatz textures with 36 samples from each texture for the experiments (Brodatz 1966). The threshold values varied from 0.2 to 0.8 along with different sample sizes from 30 to 100. In this context we also experimented with varying threshold values and constant sample size and vice versa. The results obtained for one of the textures namely D3, for both the cases are illustrated in the tables shown below. The experiment is repeated for different number of samples and threshold level pairs. In our experiments it is observed that the combination of 81 number of samples with a threshold value of 0.45 provides better classification rate. The classification rate thus obtained with these optimum values is tabulated in Table 3. Table 4 shows the classification rate obtained by setting the threshold value to 0.45 (as a middle value) and varying sample sizes, while Table 5 provides the same with 81 number of samples (found to give good results for 81 samples) and different threshold levels.

**Table 3:** Classification rate obtained for 20 brodatz textures applying DTCWPT multiresolution analysis (considering 36 samples and a threshold value of 0.65)

ID	DTCWPT ( %)	ID	DTCWPT ( %)
D3	91.1	D77	95
D6	96.1	D83	95
D20	95.8	D87	95
D21	95.3	D88	93.6
D22	95.3	D89	94
D67	95.3	D92	95
D68	95.3	D93	95.3
D71	95.3	D96	96.1
D72	93.6	D97	93.6
D73	95.3	D101	95.6

**Table 4:** Classification rate obtained for texture D3 for a threshold value of 0.45 and varrying the number of samples (applying 3 level DTCWPT multiresolution analysis)

Number of samples	Classification rate (%)
9	90
25	91.6
36	93.89
42	94.29
64	94.22
81	97.5

**Table 5:** Classification rate obtained for texture D3 by selecting 81 number of samples and varying the threshold levels (applying 3 level DTCWPT multiresolution analysis)

Threshold	Classification rate (%)
0.45	97.5
0.51	96.67
0.60	95.93
0.65	94.44
0.75	93.21

Thus further in this experiment for channel elimination purpose, we have choosen a threshold value of 0.45 for the selected 81 samples.

### Linear regression analysis for texture classification

Regression analysis is commonly used to find the relationship between two variables. The statistical models of energies of channels are used to make predictions. When a correlation coefficient shows that data is likely to be able to predict future outcomes and a scatter graph of the data appears to form a straight line, statisticians use linear regression as an option to find a predictive function. This can be modelled as

2 $$ Y_{i} \; = \;a_{0} + b_{0} X_{i} + \epsilon_{i} ,\;1 \le i \le n, $$

Here $\epsilon_{i}$ is the residue and the constant real parameters (*a*_*0*_, *b*_*0*_) are unknown. The parameters (*a, b*) are estimated by maximum likelihood, which in the case of normally independent and identically distributed errors can be estimated in terms of *X*_*i*_ and *Y*_*i*_ by least squares, i.e. to minimize

3 $$ \sum_{i = 1}^{n} \left( {Y_{i} - a - bX_{i} } \right)^{2} $$

this results in:

4 $$ \hat{b}\; = \;\frac{Sxy}{{Sx^{2} }} $$

5 $$ \hat{a} = \bar{Y} - \hat{b}\bar{X} $$

where

6 $$ \bar{X} = \frac{1}{n}\sum\limits_{i = 1}^{n} {X_{\rm i} } $$

7 $$ \bar{Y} = \frac{1}{n}\sum\limits_{i = 1}^{n} {Y_{\rm i} } $$

8 $$ Sx^{2} = \frac{1}{n - 1}\sum\limits_{i = 1}^{n} {\left( {X_{\rm i} - \bar{X} } \right)^{2} } $$

9 $$ Sy^{2} = \frac{1}{n - 1}\sum\limits_{i = 1}^{n} {\left( {Y_{\rm i} - \bar{{Y }} } \right)^{2} } $$

10 $$ \begin{aligned} \hfill \\ \hfill \\ Sxy = \frac{1}{n - 1}\sum\limits_{i = 1}^{n} {\left( {Y_{\rm i} - \bar{Y} } \right)} \left( {X_{\rm i} - \bar{X} } \right) \hfill \\ \end{aligned} $$

The predictors and residuals for the observed responses *Y*_*i*_ are given respectively by

11 $$ {\text{Predictor}}_{i} = \hat{Y}_{\rm i} = \hat{a} + \hat{b}X_{\rm i} $$

12 $$ {\text{Residual}}_{i} = \hat{\varepsilon }_{\rm i} = Y_{\rm i} - \hat{Y}_{\rm i} $$

The Mean residual sum of squares (per degree of freedom) given by

13 $$ {\overline{\upsigma ^{2}}} = {\rm MRSS} = \frac{1}{n - 2}\mathop \sum \limits_{i = 1}^{n} ( Y_{\rm i} - \hat{Y}_{\rm i} )^{2} $$

and the mean is given by

14 $$ \mu = \frac{1}{n}\mathop \sum \limits_{i = 1}^{n} ( Y_{\rm i} - \hat{Y}_{i} ) $$

Confidence intervals for estimated parameters are all based on the fact that the least squares estimates $\hat{a}$, $\hat{b}$ and the corresponding predictors of (the mean of) $Y_{{\rm i}}$ are linear combinations of the independent normally distributed variables.

In this paper in order to analyse the correlation between different frequency channels and to extract the texture features, linear regression modelling is employed. We calculated the energy values for each channel pair (total 2 values, one for each channel) and these are modelled as two random variables. It is found that relation between these two random variables can be approximated by linear regression modelling. The algorithm used to extract the texture feature is summarized as follows (Rahman et al. 2011):

[Input]: channel pair list and energy matrix E for each texture
[Output]: texture feature

1. Each of channel pairs obtained from pre-processing algorithm, form two random variables. The energy values of corresponding channel pair taken from the channel energy matrix form the values assigned to the variables.
2. Compute the regression parameters $\hat{b}$ and $\hat{a}$ of the above values using (4) and (5).
3. Calculate the Predictor $\widehat{Y}_{{\rm i}}$ using (11).
4. Estimate the variance and mean using Eqs. (13) and (14).
5. Calculate the residual between $Y_{\rm i}$ and $\hat{Y}_{{\rm i}}$ as $\left| {\hat{Y}_{\text{i}} - Y_{\text{i}} } \right|$.
6. Repeat 1–5 for each texture sample
7. Now the channel pairs, corresponding correlation coefficient, regression parameters, mean and variance, characterize the texture features. Using these, create the database containing the feature list of each texture.

## Texture classification algorithm

Classification refers to as assigning a physical object or incident into one of a set of predefined categories. In texture classification the goal is to assign an unknown sample image to one of a set of known texture classes and it is one of the four problem domains in the field of texture analysis. Texture classification process involves two phases: the learning phase and the recognition phase. In the learning phase, the target is to build a model for the texture content of each texture class present in the training data, which generally comprises of images with known class labels. The texture content of the training images is captured with the chosen texture analysis method. This yields a set of textural features for each image. These features can be scalar numbers or discrete histograms or empirical distributions. Examples are spatial structure, contrast, roughness, orientation, etc. In the recognition phase the texture content of the unknown sample is first described with the same texture analysis method. Then the textural features of the sample are compared to those of the training images with a classification algorithm, and the sample is assigned to the category with the best match. Optionally, if the best match is not sufficiently good according to some predefined criteria; the unknown sample can be rejected.

### Learning phase

The learning phase includes the pre-processing algorithm (“Correlation between frequency channels”) and the feature extraction algorithm (“Linear regression analysis for texture classification”). The outcome of pre-processing algorithm is the channel energy matrix and top channel pair list while that of the feature extraction algorithm is the feature list of the textures in the database which includes the channel pair list, correlation coefficient, mean, variance and the linear regression parameters. The learning algorithm is summarised as follows

[Input]: texture samples
[Output]: database containing texture features

1. Take different samples of the given texture, apply pre-processing algorithm (as in “Correlation between frequency channels”) and derive the energy matrix and channel pair list.
2. Select the channel pairs with correlation coefficient greater than threshold value and arrange them in the descending order of correlation coefficients.
3. Apply feature extraction algorithm (as in “Linear regression analysis for texture classification”) and form the database.
4. Repeat steps 1–3 for all textures.

According to central limit theorem under certain conditions, the sum of a large number of random variables will have an approximately normal distribution. For example if (${\text{x1}}, \ldots ,{\text{ xn}}$) is a sequence of independent and identically distributed random variables, each having mean μ and variance σ^2^, then the central limit theorem states that

15 $$ \sqrt n \left( {\frac{1}{n}\sum\limits_{i = 1}^{n} {x_{\rm i} - \upmu } } \right)\mathop{\longrightarrow}\limits_{}^{d}N\left( {0,\,\upsigma^{2} } \right) $$

For a normal distribution the probability density function is dependent on mean, μ and variance, σ and is given by

16 $$ P(x) = 1/\left( {\sigma \sqrt {2\pi } } \right)e^{{ - \frac{1}{2}\left( {\frac{x - \mu }{\sigma }} \right)^{2} }} $$

Here *P(*−*3σ* < *x* < + *3σ)* = 99.7 %, since mean $\mu \approx 0$. In this experiment the value of *x* represents the energy values of the channels corresponding to the channel pairs in the database.

### Recognition phase

In the recognition phase an unknown texture serves as the input. The classification algorithm is summarized as follows

[Input]: unknown texture
[Output]: unknown texture classified

1. Decompose the unknown texture using multiresolution analysis tool and obtain its energy vector *V*.
2. Select one texture from the database.
3. Choose the topmost channel pair.
4. Compute the regression parameters, mean and variance of the corresponding channel pair.
5. From energy vector *V* of the unknown texture, select the energies corresponding to the selected channel pair.
6. Consider one of the energy of two channels as $X_{\rm i}$ and other as $Y_{\rm i}$ and applying linear regression modelling, find predictor $\widehat{Y}_{\rm i}$, using (11).
7. Compute the residue $\left| {\hat{Y}_{\text{i}} - Y_{\text{i}} } \right|.$
8. If the residue is greater than $\upmu \pm 3\upsigma$, eliminate the texture from candidate list, choose the next texture and repeat steps 3–7.
9. Else select the next channel pair and repeat steps 4–7. If for all the channel pairs the residue is less than $\upmu  \pm 3\upsigma$, then that unknown texture is assigned to that corresponding texture class.
10. If there is only one texture left in the database, the unknown texture is assigned to that texture class.

## Experimental results

**Fig. 2:**
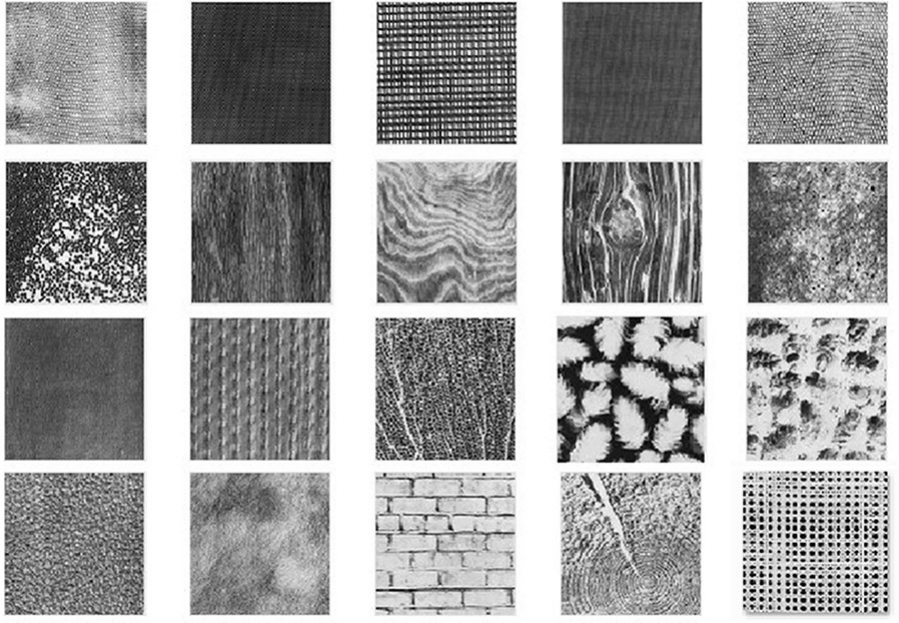
Brodatz texture images used in the experiments: from *left* to *right* and *top* to *bottom*: D3, D6, D20, D21, D22, D67, D68, D71, D72, D73, D77, D83, D87, D88, D89, D92, D93, D96, D97 and D101

The twenty textures from Brodatz database (Brodatz 1966) shown in Fig. 2 are used in the experiments. For each texture 81 samples of size 128 × 128 are formed by an overlap of 64 pixels in both horizontal and vertical directions to form a database of 1620 samples. From these, half of the samples are used for training purpose and the remaining for testing. The above mentioned multiresolution analysis tools namely the discrete wavelet transform, discrete wavelet packet transform and the dual tree complex wavelet packet transform are used in the experiments.

First we considered one level Dual tree complex wavelet packet transform (DTCWPT) as the multiresolution analysis tool. But it is found that for some textures while applying channel elimination, we will be left with little channel pairs for further processing. So two level dual tree complex wavelet packet transform (DTCWPT) is chosen. It provides 16 channels when decomposed. Energy of each of the channels is calculated using (1), and an energy vector of length 16 is formed. Thus the energy vector obtained for all the samples is arranged to form an energy matrix of size 40 × 16 as shown in Table 6. From the energy matrix, a correlation coefficient matrix is formed, which is of size 16 × 16. The (i, j)^th^ element of the matrix represents the correlation between the i and jth channels. The correlation coefficient matrix obtained for texture D3 using 2 level DTCWPT is shown in Table 7. From these, we sort the channel pairs in the descending order of the correlation coefficients (neglect the correlation values between same channels). We experimented with various threshold values from a lower boundary value of 0.2 to a higher boundary value of 0.8. For presentation we are highlighting the results obtained for texture D3. As explained in “Correlation between frequency channels”, threshold value of 0.45 is chosen and those channel pairs whose correlation coefficient is below this threshold are neglected. It is observed that for texture D3, only 41 channel pairs have correlation coefficient value above the chosen threshold, using 2 level dual tree complex wavelet packet transform decomposition. So all other channel pairs are eliminated and not listed in the database.

**Table 6:** Energy matrix obtained for texture D3 using two level DTCWPT

807	130	99	51	31	64	19	31	25	19	45	30	10	13	13	17
794	124	104	54	29	55	19	29	33	23	54	34	10	15	14	19
810	112	112	52	26	50	16	29	31	23	54	36	10	16	12	19
735	125	107	57	29	62	18	30	30	21	50	33	10	15	12	20
840	118	100	48	29	60	18	28	25	19	46	28	9	13	11	17
826	125	100	53	29	58	19	31	27	21	46	30	10	15	13	18
810	109	109	48	25	51	17	30	33	24	57	34	10	15	13	19
841	117	111	52	27	59	17	30	28	19	52	32	9	13	12	19
631	131	105	57	31	70	19	29	27	21	44	34	11	15	13	20
807	123	110	55	29	59	19	31	26	19	50	30	10	14	13	17
783	109	111	51	27	55	18	29	31	24	53	33	11	15	13	19
853	104	117	52	26	51	16	30	32	22	57	33	10	15	13	19
773	121	110	55	27	60	18	30	28	18	50	31	10	13	12	19
758	114	112	52	32	63	19	30	26	18	52	31	9	12	12	17
773	116	107	57	30	60	21	33	28	22	50	32	11	15	15	19
795	114	119	51	26	53	18	30	33	24	59	37	11	15	14	19
908	107	106	54	28	54	18	31	30	19	54	31	9	13	13	19
682	121	113	54	31	67	19	30	28	22	49	36	11	15	13	20
757	125	103	57	33	66	21	32	27	20	46	32	11	15	14	18
750	121	112	54	29	59	20	32	32	25	55	36	12	17	16	21
873	105	109	54	26	54	17	32	33	21	58	33	10	14	13	20
850	114	108	54	31	62	19	31	30	20	55	33	10	13	13	19
813	120	96	49	30	60	18	28	24	17	44	29	9	13	11	17
782	124	103	55	30	61	21	31	29	22	48	32	11	16	14	20
819	111	110	53	25	55	17	31	33	25	56	37	11	15	14	20
936	97	101	52	27	52	17	31	30	19	54	31	9	13	12	19
820	112	106	55	31	60	18	28	31	21	56	37	10	14	12	20
903	99	88	50	26	47	17	28	24	18	42	29	9	13	12	18
791	115	111	52	28	55	20	29	31	22	54	33	11	15	14	19
883	94	108	52	23	47	15	29	31	21	55	34	9	14	12	20
904	96	106	54	29	51	16	27	31	19	53	33	9	13	11	18
716	109	105	51	31	54	17	26	26	20	45	33	9	13	12	18
787	122	98	54	29	54	20	29	27	21	47	34	11	15	15	19
823	106	113	49	24	49	16	27	32	22	58	34	10	13	12	18
912	98	109	51	26	49	15	27	29	19	54	33	9	13	11	19
764	101	111	54	29	53	18	28	33	23	57	37	11	15	13	20
799	117	96	54	30	55	19	29	27	21	46	35	10	15	13	19
790	122	107	51	27	56	18	30	31	23	56	34	12	15	14	19
847	99	120	53	26	53	16	29	34	22	60	34	9	13	12	19
846	113	107	49	30	58	17	28	30	19	53	30	9	13	12	18

**Table 7:** Correlation coefficient matrix obtained for texture D3 using 2 level DTCWPT

1	−0.671	−0.141	−0.360	−0.524	−0.664	−0.519	−0.097	0.151	−0.315	0.309	−0.293	−0.545	−0.545	−0.385	−0.233
−0.671	1	−0.210	0.331	0.608	0.791	0.738	0.372	−0.402	0.005	−0.498	−0.147	0.439	0.391	0.437	−0.034
−0.141	−0.210	1	0.042	−0.310	−0.074	−0.292	0.151	0.672	0.478	0.776	0.489	0.144	0.164	0.105	0.246
−0.360	0.331	0.042	1	0.475	0.484	0.464	0.458	0.005	0.061	−0.131	0.235	0.364	0.285	0.342	0.470
−0.524	0.608	−0.310	0.475	1	0.803	0.690	0.087	−0.522	−0.326	−0.548	−0.203	0.083	0.039	0.095	−0.157
−0.664	0.791	−0.074	0.484	0.803	1	0.671	0.375	−0.406	−0.172	−0.438	−0.158	0.263	0.157	0.195	−0.003
−0.519	0.738	−0.292	0.464	0.690	0.671	1	0.506	−0.347	0.052	−0.460	−0.115	0.572	0.461	0.647	0.101
−0.097	0.372	0.151	0.458	0.087	0.375	0.506	1	0.136	0.245	0.094	0.033	0.490	0.387	0.612	0.381
0.151	−0.402	0.672	0.005	−0.522	−0.406	−0.347	0.136	1	0.727	0.904	0.683	0.310	0.339	0.301	0.551
−0.315	0.005	0.478	0.061	−0.326	−0.172	0.052	0.245	0.727	1	0.512	0.790	0.715	0.808	0.660	0.653
0.309	−0.498	0.776	−0.131	−0.548	−0.438	−0.460	0.094	0.904	0.512	1	0.529	0.071	0.053	0.103	0.300
−0.293	−0.147	0.489	0.235	−0.203	−0.158	−0.115	0.033	0.683	0.790	0.529	1	0.529	0.586	0.421	0.703
−0.545	0.439	0.144	0.364	0.083	0.263	0.572	0.490	0.310	0.715	0.071	0.529	1	0.862	0.896	0.655
−0.545	0.391	0.164	0.285	0.039	0.157	0.461	0.387	0.339	0.808	0.053	0.586	0.862	1	0.781	0.662
−0.385	0.437	0.105	0.342	0.095	0.195	0.647	0.612	0.301	0.660	0.103	0.421	0.896	0.781	1	0.555
−0.233	−0.034	0.246	0.470	−0.157	−0.003	0.101	0.381	0.551	0.653	0.300	0.703	0.655	0.662	0.555	1

**Fig. 3:**
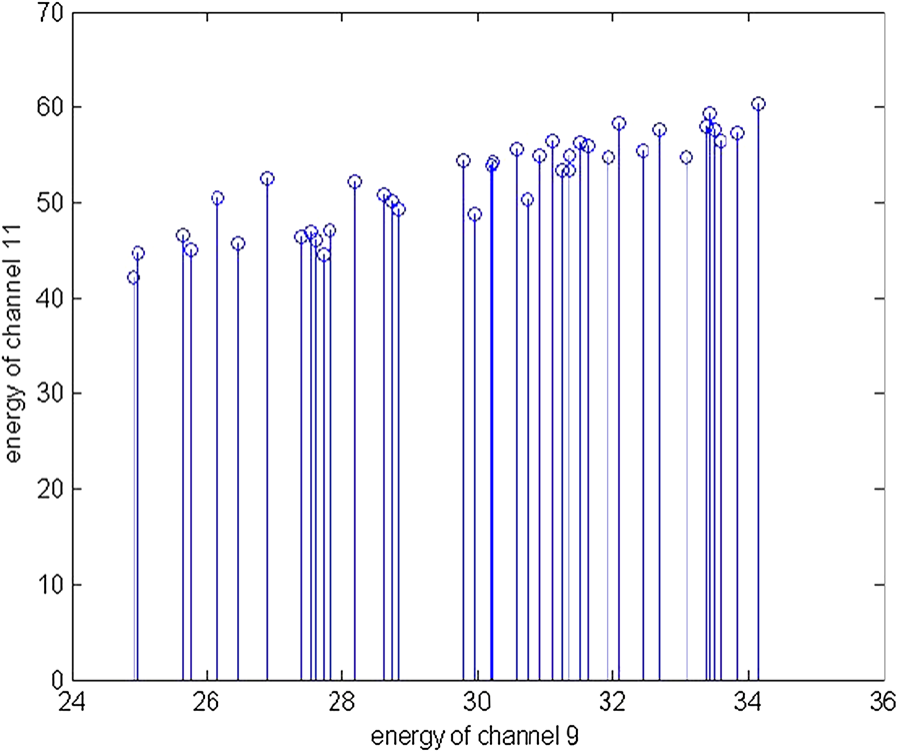
Energy distribution of channel pair of D3 having the highest correlation coefficient using 2 level DTCWPT multiresolution tool

A database is created for each of the textures, which includes the top channel pairs with correlation coefficient greater than the predefined threshold, corresponding linear regression parameters, mean and variance. The linear regression parameters for these channel pairs can be determined using (4) and (5). For top channel pairs the distribution of energy values from the energy matrix represents a straight line. Figure 3 shows the approximate linear relationship between the channel pair having the highest correlation in the case of texture D3 using two level DTCWPT as the multiresolution analysis tool. Using (14) and (15) the variance and mean is calculated and the database is created. Table 8 shows the top five channel pairs and the corresponding database values for texture D3 using two level DTCWPT.

**Table 8:** Top 5 channel pair list for texture D3 and the corresponding database values using 2 level DTCWPT

Channel pairs	Correlation coefficient	Regression parameters ‘a’ and ‘b’	Mean	Variance
9, 11	0.9040	1.6010, 4.3901	0	2.0784
13, 15	0.8969	1.1873, 0.7825	0	0.5145
13, 14	0.8622	1.1725, 2.2148	0	0.6054
10, 14	0.8087	0.4904, 4.1647	0	0.7028
5, 6	0.8032	1.8875, 2.6717	0	3.3411

In our work we have mainly focussed on three level decomposition to get better performance and the performance of three level DTCWPT is compared with that of DWPT and DWT. Discrete wavelet packet transform and dual tree complex wavelet transform produces 64 channels when three level decomposition is considered. Thus the correlation coefficient matrix is of size 64 × 64. But in the case of 3 level DWT there are only 10 channels and thus a 10 × 10 correlation coefficient matrix is obtained. In the case of DTCWPT the number of channel pairs with correlation coefficient above the predefined threshold of 0.45 is 512 for texture D3, while the same in the case of DWPT and DWT is 377 and 10 respectively. Table 9 shows the database created for texture D3 using 3 level DWT. The top 5 channel pair list for texture D3 and the corresponding database values using 3 level DTCWPT and DWPT are listed in Tables 10 and 11 respectively. It is found that for brodatz texture D101, using 3 level DTCWPT, 254 channel pairs are obtained which have correlation coefficient above 0.45.

**Table 9:** Database created for texture D3 using 3 level DWT

Channel pairs	Correlation coefficient	Regression parameters ‘a’ and ‘b’	Mean	Variance
1, 4	0.8013	2.7729, 3.3465	0	4.1292
2, 5	0.7678	1.7739, 24.4591	0	2.8747
5, 9	0.7409	2.2671, −0.7171	0	9.2205
1, 8	0.6049	5.6742, 4.1983	0	14.8954
2, 9	0.5941	4.2000, 50.4331	0	11.0436
5, 10	0.5562	1.0696, −1.1920	0	7.1715
9, 10	0.5526	0.3473, 18.0255	0	7.1920
4, 8	0.5518	1.4957, 36.2262	0	15.6003
1, 3	0.4722	0.1602, 6.6698	0	0.5964
2, 3	0.4602	0.1603, 6.6910	0	0.6007

**Table 10:** Top 5 channel pair list for texture D3 and the corresponding database values using 3 level DTCWPT

Channel pairs	Correlation coefficient	Regression parameters ‘a’ and ‘b’	Mean	Variance
21, 22	0.9185	0.5441, 7.7883	0	2.5157
36, 44	0.8919	1.2642, 0.9915	0	1.8890
37, 38	0.8903	1.1344, 0.1296	0	1.0028
38, 40	0.8854	1.0531, 2.9983	0	1.2173
33, 35	0.8764	1.1798, −0.6892	0	1.9534

**Table 11:** Top 5 channel pair list for texture D3 and the corresponding database values using 3 level DWPT

Channel pairs	Correlation coefficient	Regression parameters ‘a’ and ‘b’	Mean	Variance
8, 20	0.8494	0.3350, 1.0720	0	0.1585
3, 9	0.8458	0.3893, 0.8328	0	1.0266
20, 24	0.8375	0.9267, 0.0694	0	0.1815
6, 18	0.8344	0.4684, −1.7973	0	1.0072
8, 24	0.8206	0.3581, 0.8022	0	0.1899

**Fig. 4:**
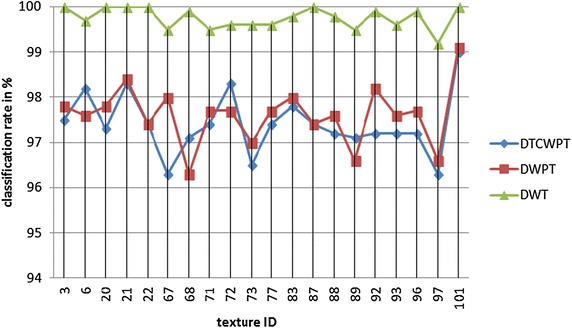
Comparison of experimental results using different multiresolution analysis

In this experiment 20 brodatz textures were taken, and each of the multiresolution analysis methods listed above is combined with the linear regression modelling method for texture classification. Figure 4 shows the classification rate for all the 20 textures using these multiresolution analysis methods.

It is noted from Fig. 4 that discrete wavelet transform provides the best texture classification rate for all the textures compared with discrete wavelet packet transform and dual tree complex wavelet packet transform. It is also observed that for some of the textures dual tree complex wavelet packet transform and discrete wavelet packet transform provides the same classification rate. Contrary to the above, it is found that for brodatz textures D6, D68, D72 and D89 dual tree complex wavelet packet provides better classification rate compared to that of discrete wavelet packet transform. In terms of the time elapsed for creating the database, it is found to be less for discrete wavelet transform. For dual tree complex wavelet packet transform the time needed for creating the database for 20 brodatz textures is 12 min, that for discrete wavelet packet transform is 5 min and 49 s while in the case of discrete wavelet transform the time expended is only 1 min and 38 s. We infer that computational complexity is also much reduced by the adoption of the multiresolution analysis tool of discrete wavelet transform. This is because of the simplicity in the application of the filters. For the DWPT and DTCWPT, applying the appropriate filter also plays a major role in the accuracy of the extracted features. Designing the filter required for the context is understood to give better response.

## Conclusion

In this paper, various multiresolution analysis tools such as discrete wavelet transform, discrete wavelet packet transform or dual tree complex wavelet packet transform is combined with linear regression modelling for classification of textures. The dual tree complex wavelet packet transform (DTCWPT) which is the most advanced version of wavelet transforms is expected to produce the best classication rate. But in our experiments the discrete wavelet transform (DWT) outperforms both the dual tree complex wavelet packet transform (DTCWPT) and the discrete wavelet packet transform (DWPT) when applied for classification of textures. This work has focused on texture classification method. Application of this method to texture segmentation may be explored in future. The performance is also dependent on the filters used. Requirement specific filter can be designed and tried for better accuracy for DWPT and DTCWPT in future.
